# Biomaterials Designed to Modulate Reactive Oxygen Species for Enhanced Bone Regeneration in Diabetic Conditions

**DOI:** 10.3390/jfb15080220

**Published:** 2024-08-08

**Authors:** Mingshan Li, Zhihe Zhao, Jianru Yi

**Affiliations:** 1 State Key Laboratory of Oral Diseases & National Clinical Research Center for Oral Diseases, West China Hospital of Stomatology, Sichuan University, Chengdu 610041, China; 2 Department of Orthodontics, West China Hospital of Stomatology, Sichuan University, Chengdu 610041, China

**Keywords:** diabetes mellitus, reactive oxygen species, biomaterials, bone regeneration

## Abstract

Diabetes mellitus, characterized by enduring hyperglycemia, precipitates oxidative stress, engendering a spectrum of complications, notably increased bone vulnerability. The genesis of reactive oxygen species (ROS), a byproduct of oxygen metabolism, instigates oxidative detriment and impairs bone metabolism in diabetic conditions. This review delves into the mechanisms of ROS generation and its impact on bone homeostasis within the context of diabetes. Furthermore, the review summarizes the cutting-edge progress in the development of ROS-neutralizing biomaterials tailored for the amelioration of diabetic osteopathy. These biomaterials are engineered to modulate ROS dynamics, thereby mitigating inflammatory responses and facilitating bone repair. Additionally, the challenges and therapeutic prospects of ROS-targeted biomaterials in clinical application of diabetic bone disease treatment is addressed.

## 1. Introduction

Diabetes represents a chronic metabolic disorder characterized by insufficient insulin production by the pancreas or impaired utilization of the produced insulin [1]. The International Diabetes Federation’s Diabetes Atlas reported a global prevalence of 10.5% among adults aged 20–79 years in 2021 [2]. China experiences a higher adult diabetes prevalence of 12.8% according to ADA standards [3]. The increasing prevalence of diabetes puts a significant burden on the public health system.

Hyperglycemia, a frequent consequence of uncontrolled diabetes, contributes to the development of an oxidative environment that can lead to significant damage across multiple bodily systems [4]. Besides the well-documented vascular damages to organs such as the heart and brain, the influences of hyperglycemia on the bone have received increasing attention. Diabetes-related bone fragility is a prevalent and serious complication. Individuals with both type 1 and type 2 diabetes face an elevated risk of fractures compared to the general population. In a meta-analysis [5], patients with diabetes exhibited a 32% higher risk of any fracture relative to patients without. Furthermore, bone remodeling and regeneration are markedly impaired within hyperglycemic conditions. This impairment culminates in an impediment to the healing of osseous injuries among diabetic patients [6].

Diabetic complications are closely linked to oxidative stress [7,8]. Reactive oxygen species (ROS), chemically active oxygen-containing molecules produced within living systems, represent natural byproducts of aerobic organisms’ oxygen metabolism [9]. These ROS include superoxide, hydroperoxyl radicals, singlet radicals, hydroxyl radicals, nitric oxide, and peroxynitrite [10]. Given the comprehensive involvement in diabetic complications, ROS have emerged as a promising therapeutic target.

Tissue engineering offers a highly promising avenue for efficient bone repair due to its ability to support cell growth, modulate cellular signals, and influence the microenvironment [11]. Recently, ROS-scavenging biomaterials have shown great potential in the field of diabetic bone repair therapy by directly or indirectly balancing ROS production and elimination to mitigate inflammation. In this review, we delve into the mechanisms underlying ROS generation and disruption of bone homeostasis in diabetes. Additionally, we summarize the current research progress related to ROS scavenging biomaterials and discuss their future prospects.

## 2. The Role of ROS in Bone Metabolism under Diabetic Physiologic Conditions

### 2.1. ROS Production

Diabetes mellitus can result in elevated accumulation of ROS, leading to heightened oxidative stress [12], which inhibits osteogenesis and promotes osteoclastogenesis, ultimately impeding bone regeneration [13]. This sub-section specifically examines the origins of ROS overproduction in hyperglycemia and the associated underlying mechanisms.

#### 2.1.1. Mitochondrial ROS Production

The mitochondrial pathway serves as the primary route for endogenous ROS production. Within this pathway, a proton gradient is established across the inner mitochondrial membrane via the electron transport chain (ETC), which consumes cytosolic oxygen and generates adenosine triphosphate (ATP) [14]. The ETC comprises four main complexes (Complexes I, II, III, and IV) that utilize NADH and FADH2 as electron donors. Ultimately, these complexes reduce oxygen to water, while protons are actively pumped into the intermembrane space, creating a voltage gradient across the inner mitochondrial membrane. The energy of this proton gradient drives ATP synthesis by ATP synthase [15].

Under normal aerobic conditions, over 90% of consumed oxygen is directly reduced to water by cytochrome oxidase in the mitochondrial intramembrane ETC via a four-electron mechanism. The remaining less than 10% undergoes reduction, forming ROS such as superoxide through a single-electron sequential pathway [16,17]. However, in diabetic conditions, excess glucose metabolism in the tricarboxylic acid cycle leads to increased NADH and FADH2 entry into the mitochondrial ETC. Consequently, a proton gradient across the inner membrane reaches its maximum threshold. This elevated ATP/ADP ratio and hyperpolarization of the inner mitochondrial membrane partially inhibit electron transfer in Complex III, resulting in electron accumulation within coenzyme Q [18,19]. In this scenario, electron transfer is hindered, and coenzyme Q exclusively provides electrons for molecular oxygen, leading to superoxide overproduction and subsequent ROS generation [20]. Additionally, morphological changes in mitochondria induced by high glucose levels have been identified as upstream factors contributing to ROS production [21,22].

#### 2.1.2. Non-Mitochondrial ROS Production

- Glyceraldehyde-3-phosphate in Glucose Autoxidation

Glyceraldehyde-3-phosphate (GAP), the phosphorylation product of glucose, undergoes oxidation by glyceraldehyde-3-phosphate dehydrogenase (GAPDH) during glycolysis. However, GAPDH activity in the pancreas diminishes in hyperglycemia, resulting in the accumulation of excess GAP [23]. This surplus GAP undergoes autoxidation, leading to the formation of hydrogen peroxide and α-ketoaldehydes [24]. Consequently, intracellular ROS increase in diabetic cells, disrupting cellular homeostasis.

Hyperglycemia leads to an excess of superoxide that overwhelms the antioxidant defense system, causing oxidative stress and damage to nuclear DNA and other biomolecules [25,26]. DNA damage activates the DNA repair enzyme poly(ADP-ribose) polymerase-1 (PARP-1), which inhibits GAPDH [27]. Consequently, elevated levels of GAP and other glycolytic intermediates, along with glucose, trigger additional pro-oxidative pathways. Notably, increased concentrations of the glycolytic metabolite fructose-6-phosphate and enhanced flux through the hexosamine pathway contribute to GAPDH inhibition, facilitating glucose accumulation [28].

- Polyol Pathway

Aldose reductase (AR) is a cytosolic NADPH-dependent oxidoreductase that catalyzes the reduction of various carbonyl compounds. It plays a crucial role as the first rate-limiting enzyme in the polyol pathway [29,30]. At low glucose concentrations, AR exhibits minimal affinity for glucose, but it becomes activated at high glucose levels, utilizing NADPH [31]. This activation leads to an increase in sorbitol levels. Sorbitol dehydrogenase further converts sorbitol to fructose. The elevated aldose reductase activity results in reduced NADPH levels. Since NADPH is essential for regenerating reduced glutathione (GSH), this reduction affects the activity of glutathione peroxidase (GPx) and the overall GSH level. Consequently, an imbalance in the NADH/NAD+ redox state (pseudohypoxia) occurs, inhibiting antioxidant defense mechanisms and leading to increased intracellular ROS [32,33]. Furthermore, high fructose levels can be phosphorylated to fructose-3-phosphate, which subsequently decomposes to 3-deoxyglucose. Both compounds serve as precursors and participate in the formation of advanced glycation end products (AGEs) through glycation [34].

- PKC Pathway

Protein kinase C (PKC) comprises a family of at least 11 distinct subtypes expressed across mammalian tissues. PKC plays a pivotal role in phosphorylating various target proteins. The conventional PKC subtype’s functionality relies on dual calcium ions and phosphatidylserine, with diacylglycerol (DAG) significantly enhancing its activity [35].

Simultaneously, elevated PKC pathway activity has been associated with the stimulation of ROS-generating enzymes, such as NADPH oxidase and lipoxygenase, collectively exacerbating cellular oxidative stress [36]. As a proximal regulator of NADPH oxidase (Nox), PKC significantly influences the conglomeration and subsequent activation of Nox1-3 subtypes. Within the cytosolic domain, Nox1-3 subtypes coalesce to form catalytically active subunits, including p47phox, through a phosphorylation-dependent process, ultimately leading to NADPH oxidase activation [37]. Additionally, PKC activates the Na ± H^+^ exchanger (NHE). This vulnerability to high glucose occurs via the PKC pathway, resulting in NHE activation. The subsequent increase in intracellular calcium concentration ([Ca^2+^]i) leads to the phosphorylation of calcium/calmodulin-dependent protein kinase II (CaMKII), which further activates NADPH oxidase, resulting in increased ROS. Furthermore, the interaction between AGEs and their extracellular receptor (RAGE) also activates the PKC pathway and its subtypes [38].

- Hexosamine Pathway

High glucose-mediated mitochondrial superoxide overproduction inhibits GAPDH activity. Concurrently, an augmented flux of phosphorylated glucose instigates the activation of the hexosamine pathway [39]. It has been demonstrated that the hexosamine pathway can mediate the effects of ROS-induced hyperglycemic complications [40]. Activation of the hexosamine pathway leads to excessive production of high amino sugar glucosamine, which further stimulates mitochondrial ROS production, impairing mitochondrial respiration. This exacerbates oxidative stress, increases vascular permeability, and promotes angiogenesis. Additionally, the hexosamine pathway upregulates genes involved in diabetic complications by enhancing Sp1 glycosylation, leading to the activation of genes contributing to diabetic pathogenesis [39].

- Advanced Glycation End Products (AGEs)

The formation of AGEs primarily results from non-enzymatic glycation reactions between extracellular proteins and glucose [41]. In hyperglycemic conditions, glucose undergoes auto-oxidation, leading to the production of carbonyl compounds such as glyoxal, a precursor of AGEs. Additionally, glucose metabolites like GAP and dihydroxyacetone phosphate undergo non-enzymatic dephosphorylation, yielding methylglyoxal, another AGE precursor. A third precursor, 3-deoxyglucosone, forms through the cleavage of glucose-derived adducts of lysine, commonly known as Amadori products [42]. The interaction between AGEs and RAGEs activates NADPH oxidase, thereby enhancing intracellular ROS production [43]. AGEs also stimulate ROS production through the mitochondrial ETC [44]. Furthermore, the enhanced ROS concentrations contribute to AGE formation and RAGE expression, forming a vicious circle and exacerbating AGE-mediated damage [45,46].

### 2.2. ROS on Bone Remodeling in Hyperglycemia

**Figure 1:**
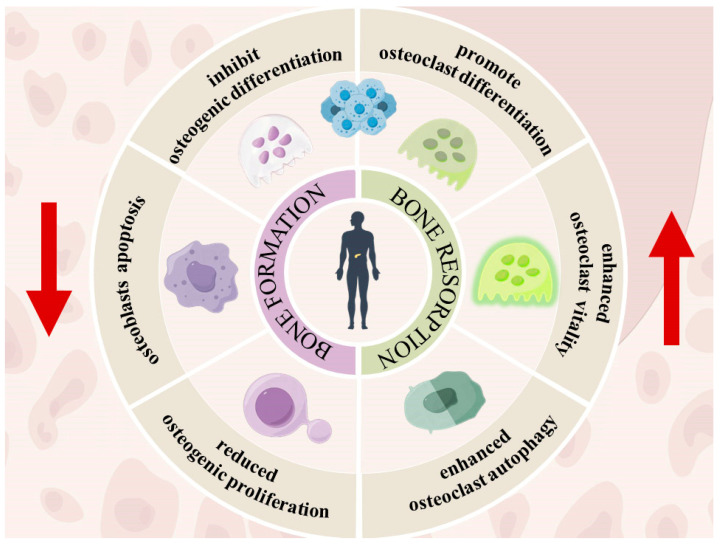
Out-of-balance bone regeneration in diabetic states. The left half depicts the inhibitory effects of diabetic condition on bone formation. The right half highlights the promotion of bone resorption.

Under physiological conditions, ROS are integral to the equilibrium between bone formation and resorption, thereby preserving skeletal integrity [47]. Nevertheless, this intricate balance is perturbed by the overproduction of ROS in diabetic states, culminating in impaired bone repair [48]. A vivid depiction of the imbalance between bone formation and bone resorption under diabetic physiologic conditions is presented in Figure 1.

Elevated levels of ROS reduce osteoblast abundance and impair their function, resulting in compromised bone formation by regulating the cell cycle. Extensive evidence suggests that oxidative stress disrupts various cellular processes involved in the differentiation of mesenchymal stem cells (MSCs), thereby negatively affecting their osteogenic potential [49,50,51,52,53]. During MSC osteogenic differentiation, oxidative stress suppresses protein kinase activation and the expression of osteogenic genes, including RUNX2 [50,54]. It has also been demonstrated that ROS overproduction activated mitochondrial apoptosis, resulting in a reduction in osteogenic differentiation and bone loss in hyperglycemia [55]. Additionally, heightened ROS levels activate signaling pathways that regulate apoptotic genes, such as the JNK/c-jun and ERK signaling cascades, ultimately promoting apoptosis in mature osteoblasts [56,57]. Endocrine disorders are common in patients with diabetes, particularly characterized by a deficiency in insulin and insulin-like growth factor-1 (IGF-1), which leads to insufficient collagen synthesis and poor bone mineralization. The chronic hyperglycemia observed in diabetes generates ROS, and levels of ROS above the critical threshold can result in β-cell dysfunction and/or death, reducing insulin secretion [58,59]. β-cells are highly sensitive to oxidative stress due to their low antioxidant defense capabilities [60].

Insulin and IGF-1 are anabolic hormones, and insulin exerts its osteogenic effects by binding to insulin receptors on the surface of osteoblasts. These effects are primarily mediated through insulin receptor substrate (IRS)-1 and IRS-2, which enhance osteoblast proliferation [61,62]. Insulin deficiency can lead to impaired bone matrix maturation and conversion, increased bone matrix degradation, and elevated bone loss. Endocrine dysfunction further deteriorates bone mass and quality, increasing the risk of osteoporosis.

Conversely, high glucose-induced ROS production increases the expression of RAGEs, contributing to osteoclast formation [45,63]. Elevated ROS levels induce receptor activator of nuclear factor-κB ligand (RANKL) expression, which stimulates osteoclastogenesis via FOXO1 production [64,65]. Furthermore, autophagy in osteoclasts induced by oxidative stress promotes bone resorption through the ROS/ER and ROS/TGEB pathways [66,67]. It is also noteworthy that RANKL-stimulated NF-κB ligand increases ROS production in MSCs, thereby enhancing osteoclast differentiation. Conversely, exposure to the antioxidant N-acetylcysteine (NAC) has been shown to inhibit MSCs’ response to RANKL, involving ROS production and osteoclastogenesis [68,69].

## 3. Chemical Compounds and Biomaterials as Antioxidant Agents

Antioxidants are agents that safeguard cellular integrity against the deleterious impacts of free radicals [70]. These substances can mitigate the detrimental consequences of oxidative stress either through direct neutralization of free radicals or via indirect mechanisms, such as the suppression of enzymatic activities responsible for free radical generation or the augmentation of endogenous antioxidant enzymatic functions. Based upon their provenance, antioxidants are principally categorized into two distinct classes: natural and synthetic.

### 3.1. Natural

Natural antioxidants are bifurcated into two principal classifications: endogenous and exogenous antioxidants. Endogenous antioxidants are implicated in the amelioration of cellular damage attributable to free radicals, initiating intrinsic cellular regeneration. Conversely, exogenous antioxidants contribute to the rectification of cellular impairments engendered by free radicals, predominantly through the stimulation of cellular regeneration, rather than its initiation.

#### 3.1.1. Endogenous

Endogenous antioxidants are mainly classified into enzymatic and non-enzymatic types [71]. The enzymatic cohort include superoxide dismutase (SOD), catalase (CAT), and glutathione peroxidase (GPx) [72]. Within the human physiological milieu, SOD manifests in three isoforms: cytoplasmic CuZn-SOD (SOD1), mitochondrial Mn-SOD (SOD2), and extracellular EC-SOD (SOD3). SOD epitomizes the initial defense mechanism in the ROS neutralization cascade. Upon activation, these enzymes expedite the dismutation of superoxide radicals into molecular oxygen and hydrogen peroxide (H_2_O_2_), thereby transmuting highly reactive ROS into less deleterious entities. Concurrently, CAT addresses the H_2_O_2_ by catalyzing its decomposition into molecular oxygen and water across diverse biological contexts [73,74]. GPx enzymes mainly exert their antioxidant prowess by facilitating the conversion of H_2_O_2_ into water, utilizing glutathione (GSH) as a reductant [75].

The non-enzymatic antioxidant representatives, such as GSH and alpha-lipoic acid, play pivotal roles in the sustenance of the cellular redox equilibrium, thereby constituting integral components of the cellular antioxidant apparatus. The cysteine amino acid, endowed with a thiol group, acts as a reductant and partakes in periodic reversible redox reactions. Cellular homeostasis of reduced glutathione is maintained via the enzymatic action of glutathione reductase (GR). GSH, in turn, is capable of reducing other enzymes and metabolites [33]. Alpha-lipoic acid, along with its reduced form dihydrolipoic acid, exhibits intrinsic free radical scavenging attributes, and supplementation with alpha-lipoic acid has been evidenced to attenuate oxidative stress and ameliorate the levels of other antioxidants [76]. Furthermore, research indicates that uric acid enhances antioxidant defenses in the pulmonary system by scavenging hydroxyl radicals (HO) and hypochlorous acid (HOCl) [77].

#### 3.1.2. Exogenous

Exogenous antioxidants such as vitamins, trace minerals, carotenoids, and polyphenols facilitate the neutralization of ROS through the activation of endogenous antioxidant enzymes or by terminating oxidative chain reactions. For example, vitamin D mitigates the activity of NADPH oxidase (NOX) and augments the activity of antioxidant enzymes, thereby expediting the elimination of ROS [78]. Ascorbic acid is known to modulate both NOXs and xanthine oxidase (XO) systems, thereby regulating ROS levels and enhancing the efficacy of other antioxidants [79]. Vitamin E, functioning as a peroxide radical scavenger, disrupts chain reactions by donating its phenolic hydrogen to peroxide radicals, resulting in the formation of non-reactive tocopherol radical species and consequently arresting oxidative chain reactions [80]. Furthermore, zinc exerts its antioxidant influence by inhibiting NOX-2 [81]. Curcumin, a naturally occurring potent antioxidant, exhibits significant hydrogen-donating antioxidant capacity, attributable to the phenolic constituents within its molecular structure [82,83]. Supplementation with the dietary polyphenol curcumin has been documented to ameliorate oxidative stress markers in individuals with metabolic syndrome (MetS) [84]. Docosahexaenoic acid (DHA), an omega-3 long-chain polyunsaturated fatty acid, possesses inherent antioxidant characteristics and has been reported for its role in diminishing mitochondrial depolarization and bolstering antioxidant defenses [85]. Additionally, DHA has been demonstrated to facilitate diabetic bone regeneration by rectifying aberrant mineralized proteins, reducing hydroxyapatite crystal mass, and mitigating oxidative stress induced by elevated glucose levels.

### 3.2. Synthetic

#### 3.2.1. Small Molecules Chemical Compounds

Butylated hydroxyanisole (BHA), butylated hydroxytoluene (BHT), propyl gallate (PG), tert-butylhydroquinone (TBHQ), and ethylenediaminetetraacetic acid (EDTA) are the most commonly used synthetic antioxidants [70]. Synthetic antioxidants, which exhibit bioequivalence to natural antioxidants, demonstrate superior potency consistent antioxidative efficacy. Nevertheless, concerns regarding their potential carcinogenic effects have constrained their application [86].

#### 3.2.2. Macromolecular Antioxidant Biomaterials

The burgeoning field of tissue regeneration engineering has seen extensive application of various biomaterials targeting ROS therapies—including scaffolds, coatings, nanoparticles, and hydrogels—in diabetic bone regeneration.

##### Scaffold

Bone, characterized by its intricate structure and diverse mechanical properties, necessitates consideration of porosity, mechanical strength, and biocompatibility in the fabrication of scaffolds for regenerative repair [87,88]. Porous materials enhance cellular and protein infiltration, thereby promoting osteoinduction, while mechanical robustness shields against external forces [89]. Materials targeting ROS can mitigate oxidative stress, either directly or indirectly, thus supporting osteogenesis in compromised tissues. Table 1 summarizes contemporary scaffolds designed for bone healing in oxidative environments employing hybrid molecules, enzyme-catalyzed agents, or click chemistry for ROS removal [90,91,92,93].

Hybrid molecular scaffolds, serving as local drug delivery systems composed of biodegradable substances, have been shown to significantly improve drug bioavailability. Zhang et al. developed α-lipoic acid (ALA)-loaded poly (lactic acid–glycolic acid copolymerization) (PLGA) microspheres using emulsion solvent evaporation and found this scaffold capable of attenuating ROS-induced dysfunction in BMSCs and achieving more effective osteogenesis in diabetic conditions [90]. Lin et al. created dAsp3-PEG-PLGA (APP) nanoparticles to load simvastatin to treat osteoporosis [94]. Polydopamine (PDA), a synthetic melanin-like polymer, exhibits exceptional adhesion properties and facilitates the attachment of various compounds to its surface [95]. PDA produces effective drug loading through metal chelation, hydrogen bonding, electrostatic interactions, and other mechanisms [96]. PDA nanoparticles are distinguished by their biocompatibility, biodegradability, and hydrophilicity, and are known to exert significant antioxidant effects, particularly against hydroxyl radicals [97]. Li et al. developed a PDA-mediated scaffold incorporating graphene oxide and hydroxyapatite nanoparticles, which expedited periodontal bone regeneration by modulating the diabetic inflammatory microenvironment and promoting osteogenesis-related cytokine secretion [92]. Reduced GSH, a classical antioxidant, functions as a co-substrate for GSH peroxidase, facilitating the reduction of peroxides and the formation of GSH disulfide [98]. Recent studies have employed glutathione as a covalent grafting agent in the development of antioxidant hydrogels, which have been 3D printed as tissue-engineered scaffolds, demonstrating their ability to scavenge ROS and promote osteoblast proliferation and differentiation through the PI3K/Akt signaling pathway, thereby accelerating bone repair in diabetic conditions [99]. Cerium oxide nanoparticles exhibit remarkable surface catalytic activity, mimicking multi-enzymatic properties and enabling manipulation of their dual valence state (Ce^4+^/Ce^3+^) under oxidative stress conditions to facilitate self-regenerative redox reactions [91]. Hence, nanoceria scaffolds represent a promising avenue for drug-free and cell-free diabetic bone repair treatments [100].

Click chemistry, renowned for its high reaction efficiency and selectivity, has garnered attention in the development of polymeric scaffolds for tissue engineering [101]. Thiol–alkene networks, in particular, are utilized for their antioxidant properties, with the inherent capacity to neutralize free radical oxygen and produce sulfoxide and sulfone groups [102]. These networks’ unique characteristics render them suitable for bone grafts in patients experiencing high oxidative stress. Touchet TJ et al. initially proposed the use of thiol–alkene biomaterials for diabetic bone grafts in 2022 [103] and later reported the fabrication of interconnected porous scaffolds with an average porosity of 97.0% in 2024 [104]. Both scaffolds effectively isolated pathological ROS levels by consuming hydrogen peroxide, with the latter having a more pronounced effect on bone infiltration and neovascularization.

**Table 1:** Summary of scaffold-based strategies targeting ROS.

Antioxidant Biomaterial	Study Design	Targeted Model	ROS Scavenger Compounds	Ref.
ALA-loaded poly (lactic-co-glycolic acid) (PLGA) microspheres	In vitro/in vivo	BMSC/STZ-induced diabetic SD rats	α-lipoic acid	[90]
PGO-PHA-AG scaffold	In vitro/in vivo	BMSC/STZ-induced diabetic SD rats‘ mandibular periodontitis	Polydopamine	[92]
Three-dimensionally printed reduced glutathione grafted gelatine methacrylate (GelMA-g-GSH)	In vitro/in vivo	MC3T3-E1/Calvarium bone defects in bone defect SD rats	GSH	[99]
nCe-scaffold	In vitro/in vivo	MSC/calvarium bone defects in bone defect SD rats and STZ-induced diabetic SD rats	Nanoceria (Ce)	[100]
Thiol–methacrylate networks	In vitro	NA ^1^	Thiol–methacrylate networks	[103]
Thiol–methacrylate interconnected porous scaffolds using emulsion templating	In vitro/in vivo	Bilateral calvarial defect model in Zucker diabetic fatty rats	Thiol–methacrylate networks	[104]

^1^ not mentioned in reference.

##### Coating

Medical bioscaffolds, implanted for their robust support strength, have found extensive clinical utility in dental implants and arthroplasty. Nonetheless, unmodified implants composed of polyetheretherketone (PEEK) and titanium struggle to navigate the multifaceted microenvironment of diabetic patients, owing to their bioinert nature, stress shielding effects, and absence of antimicrobial properties. Additionally, the surface roughness of implants influences osseointegration. Research by Boyan et al. indicated an inverse relationship between the viability of cells on a material’s surface and its roughness [105], with rougher surfaces fostering in vivo bone formation and smoother surfaces predisposing to fibrous interfaces [106,107]. It has also been reported that rough surfaces induce inflammatory cytokine expression and are prone to degradation byproducts that attract inflammatory cells and generate ROS [108]. Consequently, the development of antioxidant-functionalized implants through surface modification has emerged as a promising research direction to enhance the success rate of implantation in diabetic bone defects, focusing primarily on material-based surface modification and targeted enhancement of the coating itself. Table 2 summarizes the coating-based strategies targeting ROS.

- Material-Based Surface Modification

Commercial titanium implants are commonly subjected to surface modification techniques such as sandblasting and acid etching, which create a micro-rough morphology that increases surface area and promotes cell adhesion [109]. However, these techniques can also initiate fatigue crack nucleation and propagation, thereby compromising mechanical properties and osteoblast proliferation [110]. Dong et al. investigated the use of a phytic acid (PA) and calcium hydroxide mixture as a coating, finding that Ca-PA-modified sandblasted, large-grit, acid-etched (SLA) titanium surfaces reduced ROS production, mitigated oxidative stress damage, and promoted osteogenic differentiation BMSCs in a high-glucose environment [111]. Furthermore, the design optimization of coatings has considered alterations to the fundamental structure, such as the use of TiO_2_ nanotubes (TNTs) prepared by anodic oxidation, which mimic bone’s basic nanoscale structure and exhibit excellent biocompatibility and osseointegration [112]. Previous studies have found that the roughness of TNT surfaces is significantly less than that of SLA surfaces and slightly higher than that of mechanically polished surfaces [113]. Studies have shown that TNT-modified surfaces facilitate better cellular adhesion and proliferation, enhance alkaline phosphatase activity, and improve bone mineralization [114,115,116] in hyperglycemia. Additionally, TNT surfaces have been found to balance ROS expression by generating higher total superoxide dismutase levels under diabetic conditions, thereby providing better antioxidant capacity and attenuating osteogenesis inhibition in high glucose environments [116]. Huang et al. suggested that the increased antioxidant properties of TNT might be due to the activation of the nuclear factor-E2-related factor 2 (Nrf2)/Kelch-like ECH-associated protein 1 (Keap1)-antioxidant response element signaling pathway or the Rho-/Rho kinase (ROCK) signaling pathway [117].

- Composite Coating Optimization

It has been reported that metal ion or hydroxyapatite-modified coatings can confer antimicrobial activity and osteogenic properties to polymer scaffolds [118,119]. Wang et al. developed a multifunctional PEEK orthopedic implant modified with metal ions, incorporating dual nutrient elements of zirconium and strontium to influence mitochondrial dynamics and function, suppress the overexpression of the Drp1 gene, and reduce cellular ROS expression, thus enhancing osteogenic differentiation and osseointegration in a diabetic rat bone defect model [120]. Nanohydroxyapatite (nHA), a principal inorganic component of bone tissue, continues to be a focal point for bioactive calcium phosphate coating development due to its osteoconductive and osteoinductive advantages [121]. However, nHA’s limited mechanical properties and toughness present challenges in complex biological environments such as oxidative stress [122]. To address this, nHA has been combined with antioxidants, growth factors, or other biomaterials to enhance bone regeneration [123]. Yu et al. assembled HA-functionalized nPDA (HA/nPDA) coatings by incorporating PDA nanoparticles, which possess strong ROS-scavenging abilities, thus improving nHA’s osteogenic ability in pathological conditions like diabetes [124]. Tao et al. utilized silymarin-modified hydroxyapatite coatings to regulate oxidative stress and activate the osteogenic differentiation process in hyperglycemia through the SIRT1/SOD2 signaling pathway [125]. Ma et al. created a silk fibroin/hydroxyapatite (SF/HA) hybrid coating that significantly improved osseointegration impaired by diabetes by reactivating the ROS-mediated PI3K/Akt signaling pathway [126].

Chitosan, a deacetylated derivative of chitin, is being explored as a coating material for orthopedic implants to enhance osseointegration and cell attachment due to its minimal foreign body reaction, favorable wettability, suitable degradation rate, and versatility in forming various geometries [127]. More importantly, chitosan exhibits antioxidant and free radical scavenging activities [128,129]. It has been demonstrated that chitosan coatings can reverse osteoblast dysfunction by scavenging ROS through the reactivation of the PI3K/AKT pathway, stimulating AKT phosphorylation [130]. Ma et al. combined the osseointegration capabilities of HA and constructed a nano HA/chitosan (HA/CS) composite coating to promote osteoblast adhesion and differentiation through the reactivation of the FAK-BMP-2/Smad pathway under diabetic conditions [131].

**Table 2:** Summary of coating-based strategies targeting ROS.

Antioxidant Biomaterial	Study Design	Targeted Model	ROS Scavenger Compounds	Ref.
Ca-PA-modified SLA titanium surface	In vitro/In vivo	hBMSC/STZ-induced diabetic SD rats	Ca–phytic acid	[111]
Zn&Sr- Sulfonated PEEK	In vitro/In vivo	MC3TE-E1/STZ-induced diabetic SD rats	Zn and Sr ion	[120]
Three-dimensional coating of hydroxyapatite-functionalized nanoparticles of polydopamine (HA/nPDAs)	In vitro	MC3T3-E1	nPDA	[124]
Silibinin-modified hydroxyapatite coating	In vitro/In vivo	MC3TE-E1/STZ-induced diabetic SD rats	Silibinin	[125]
Silk fibroin-based hydroxyapatite (SF/HA) hybrid coating	In vitro/In vivo	Primary rabbit osteoblasts/diabetic rabbits	SHT	[126]
HA/CS composite coating	In vitro/In vivo	Primary rat osteoblasts/STZ-induced diabetic sheep	Nano-HA/CS composite coating	[131]

##### Nanoparticles

Nanoparticles composed of inorganic ions are characterized by their substantial surface area-to-volume ratios and distinctive physicochemical attributes, which confer pronounced biological activities even at relatively modest concentrations [132]. These nanoparticles can be homogeneously distributed with precise control over size through straightforward chemical, physical, or biological methodologies [133]. Furthermore, their mechanical robustness and the facility for surface functionalization serve as critical underpinnings for biomedical utilizations, circumventing issues such as intraventricular coalescence and indiscriminate protein binding [134]. Table 3 summarizes nanoparticles targeting ROS scavenging strategies.

Selenium is pivotal in the modulation of redox equilibrium and is indispensable for the growth and maturation of osteoblasts and osteoclasts [135]. In addition, a selenium at optimal concentrations augments antioxidant levels within BMSCs and promotes bone remodeling [136,137]. Selenium nanoparticles have been demonstrated to activate the BMP-2/β-catenin signaling cascade, thereby promoting osteogenesis through the induction of RUNX2 expression under hyperglycemic conditions, concurrently diminishing levels of ROS [138]. In addition to single elements, the catalytic capabilities of redox self-cycling in composite ions have also been the focus of research. The antioxidative potential of cerium dioxide (CeO_2_) has been identified in multiple investigations, with the mechanism involving the formation of additional oxygen vacancies to counterbalance the escalation of ROS [139,140]. Hu et al. enhanced the antioxidative properties of CeO_2_ by doping it with lanthanum ions (La^3+^), thereby modulating the concentration of oxygen vacancies within the CeO_2_ structure [139]. The group with 30% La-doped cerium nanoparticles exhibited upregulation of superoxide dismutase 1 and catalase, suppression of ROS generation, and superior osteogenic capability in diabetic rats [139].

The deployment of nanoparticles for gene delivery represents an innovative approach to ameliorate diabetic osseointegration. Chitosan gold nanoparticles conjugated with PPARγ cDNA have been shown to curtail the production of pathogenic H_2_O_2_ and nitric oxide (NO), thereby directly mitigating the oxidative stress condition [141]. Concurrently, PPARγ robustly initiates mitochondrial biogenesis and cellular viability through the phosphorylated AMP-activated protein kinase (p-AMK) and Wnt/β-catenin signaling pathways, thus consistently enhancing bone formation in diabetic conditions [141]. In addition, tetrahedral framework nucleic acids (tFNAs) are nanomaterials capable of self-assembly from four single-stranded DNA (ssDNA) molecules, regulated by temperature [142]. Exhibiting exceptional biocompatibility, safety, editability, and stability, tFNAs have been previously shown to effectively deliver oligonucleotides, such as small interfering RNA (siRNA) and microRNA [143,144], and to traverse phospholipid barriers. They also hold potential for mitigating type 2 diabetes mellitus through the phosphatidylinositol 3-kinase (PI3K)/Akt pathway [145]. Curcumin-loaded tetrahedral framework nucleic acid (tFNA) particles targeting ferroptosis have been engineered to efficaciously neutralize ROS and regulate lipid peroxidation [146]. However, there is a paucity of research integrating this nanomaterial into diabetic bone repair strategies. Hence, employing tFNAs to deliver biomaterials that modulate oxidative homeostasis and osseointegration could represent a novel research trajectory.

**Table 3:** Summary of nanoparticle-based strategies targeting ROS.

Antioxidant Biomaterial	Study Design	Targeted Model	ROS Scavenger Compounds	Ref.
La-CNPs	In vitro/in vivo	MC3TE-E1/STZ-induced diabetic SD rats	Lanthanum	[139]
Chitosan gold nanoparticles conjugated with PPARγ cDNA	In vitro/in vivo	MC3TE-E1/STZ-induced diabetic SD rats	Chitosan gold nanoparticles conjugated with PPARγ cDNA	[141]
Curcumin-loaded tetrahedral framework nucleic acid (tFNA) particle	In vitro/in vivo	BMSC/diabetic osteoporosis mice using HFD + STZ	Curcumin	[146]

##### Hydrogel

Hydrogels are garnering considerable interest within the domain of bone regeneration due to their emulation of the extracellular matrix (ECM) and provision of a conducive hydrophilic three-dimensional microenvironment for the proliferation of endogenous cells [147]. Table 4 summarizes hydrogel targeting ROS scavenging strategies.

Gelatin methacrylate (GelMA), a semi-synthetic hydrogel, is extensively employed in tissue regeneration engineering owing to its high biocompatibility and porous structure, which facilitates the unimpeded exchange of substances and supports vascularization [148]. Based on this, Liao et al. incorporated molybdenum-based polyoxometalate nanoclusters (POM) into GelMA, leveraging their pH-sensitive attributes to modulate ROS and alleviate oxidative stress, while concurrently activating the PI3K/Akt signaling pathway to enhance bone regeneration in diabetes [149]. Similarly, Wang et al. introduced glutathione (GSH) into GelMA, demonstrating that GelMA-g-GSH diminished ROS levels and fostered osteogenic differentiation predominantly through the activation of the PI3K/Akt signaling pathway in hyperglycemia [99].

The incorporation of pharmacological agents to curtail ROS represents a novel avenue of research. Metformin, a primary medication for hyperglycemia management, not only directly lowers blood glucose levels but also impedes ROS generation in mitochondrial Complex I and shields against mitochondrial fission resultant from hyperglycemic conditions [150,151]. Moreover, metformin stimulates bone formation by engaging bone-related pathways such as AMPK, ERK1/2, ATK, and Wnt pathways [152]. Consequently, metformin addresses the triad of high glucose, excessive ROS, and mitochondrial dysfunction within the diabetic milieu [150]. Lao et al. devised a multifaceted composite hydrogel by amalgamating metformin-loaded zeolitic imidazolate framework-8 (ZIF-8) nanoparticles with GelMA, effectively disrupting the cycle of chronic inflammation and elevated ROS that precipitate bone anomalies in diabetes, thereby aiding bone regeneration [153].

The encapsulation of critical bioactive molecules within hydrogel matrices have also been explored. A recent study synthesized a composite logistic hydrogel, incorporating interleukin 10 (IL-10) and bone morphogenetic protein-2 (BMP-2) [154]. This hydrogel was specifically formulated for the controlled delivery of agents in response to fluctuations in glucose levels and ROS. The application of BMP-2 is posited to elicit a response from IL-10—which in turn augments the endogenous antioxidant mechanisms, thereby attenuating oxidative stress during significant elevations in ROS—and facilitate diabetic bone regeneration [154]. In parallel, Wang et al. reported the development of a mesoporous silica nanoparticle-integrated PDLLA (poly(dl-lactide))-PEG-PDLLA (PPP) thermosensitive hydrogel. This hydrogel, laden with stromal cell-derived factor 1 (SDF-1), is engineered to modulate the diabetic microenvironment and enhance osteogenesis. It achieves this by reactivating the AMPK/β-catenin signaling cascade, thus mitigating the suppression of osteogenic differentiation in BMSCs [155].

**Table 4:** Summary of hydrogel-based strategies targeting ROS.

Antioxidant Biomaterial	Study Design	Targeted Model	ROS Scavenger Compounds	Ref.
GelMA/POM nano-hydrogel	In vitro/In vivo	MC3TE-E1/STZ-induced diabetic SD rats	Mo-based polyoxometalate nanoclusters (POM)	[149]
Three-dimensionally printed reduced glutathione grafted gelatine methacrylate (GelMA-g-GSH)	In vitro/In vivo	MC3T3-E1/calvarium bone defects in bone defect SD rats	GSH	[99]
Metformin-loaded zeolitic imidazolate frameworks nanoparticle-modified hydrogel(GelMA/Met@ZIF-8)	In vitro/In vivo	BMDMs and MC3T3-E2/calvarium bone defects in bone defect SD rats	Metformin	[153]
a double-network hydrogel consisting of phenylboronic-acid-crosslinked poly(vinyl alcohol) and gelatin colloids	In vitro/In vivo	BMDMs and MC3T3-E1/calvarium bone defects in bone defect SD rats	BMP-2 (HIB)	[154].
PDLLA-PEG-PDLLA-Met@MSN-SDF-1 hydrogel	In vitro/In vivo	rBMSCs/STZ-induced diabetic rats	Met@MSN (metformin)	[155]

## 4. Future and Prospects

The amelioration of diabetic bone regeneration through the scavenging of ROS is a promising therapeutic avenue. Nevertheless, this approach necessitates a multifaceted strategy encompassing development, research, and clinical translation to address the challenges and critical issues inherent in treating the disease with ROS-scavenging materials. Significantly, ROS are pivotal to normal cellular physiological processes, serving as the primary defense against bacterial invasion and acting as a vital secondary messenger in numerous signaling cascades [156]. Consequently, the objective is not the complete eradication of ROS but rather the moderation of their excess to prevent detrimental effects on cellular functions while preserving the physiological concentrations that confer benefits.

Currently, there is insufficient evidence to reach a definitive consensus on antioxidant therapy as a one-stop solution for improving human insulin and glucose regulation. The effectiveness of antioxidant therapy is likely influenced by the treatment environment, due to significant differences in antioxidant treatment protocols and study populations, as well as the recognized physiological production of ROS [157]. However, it is well established that antioxidant therapy is clinically effective in treating diabetes and its complications, and antioxidants should be part of the therapeutic regimen [158,159]. Despite extensive research on the efficacy of antioxidants in type 1 diabetes and type 2 diabetes, which shows that some antioxidant biomaterials can improve insulin resistance and counteract endocrine disorders, the poor efficacy of standalone antioxidant supplements may be due to their solubility, permeability, stability, and specificity issues [160]. Therefore, modern therapeutic strategies for diabetes should include the development of new delivery methods and antioxidants with more stable physicochemical properties, such as scaffolds, coatings, nanoparticles, hydrogels, and so on.

The synthesis of biomaterials dedicated to the removal of ROS demands meticulous consideration of the entire process. Such materials must either inherently possess ROS-scavenging properties or be expertly impregnated with them, in addition to promoting osteogenesis. This requirement imposes specific criteria on their physical attributes. Given the intricate architecture and the varied mechanical characteristics and dynamics of bone, artificial bone constructs targeting ROS must exhibit mechanical robustness commensurate with the growth demands of the natural bone matrix. Concurrently, the porous nature of bone—a salient feature—should be emulated in the design of artificial materials, with microporous structures of appropriate pore diameters to support cellular attachment, proliferation, migration, and enhanced osteogenic differentiation [11]. Moreover, the adaptability of these biomaterials is crucial. In the context of the extracellular matrix’s complexity under hyperglycemic conditions, antioxidants that are tailored to the spatial, temporal, and dosage specifications of the local microenvironment are more likely to yield superior therapeutic outcomes. The integration of multi-responsive materials, capable of dynamically recognizing and responding to the microenvironment, is imperative [161]. These materials should be designed to react precisely to various stimuli, including enzymes, temperature, pH, and light. Furthermore, the capacity for feedback is an emerging direction in the development of ROS-targeting biomaterials. The mere incorporation of suitable materials does not constitute a definitive solution; rather, their combination with intelligent biosensors or devices can enable clinicians to monitor material degradation or therapeutic efficacy in real time, thereby facilitating the customization of treatment protocols. To expedite their clinical application, rigorous clinical investigations exploring the long-term biological impacts of ROS-scavenging biomaterials on animal and human subjects are indispensable [162].

## Data Availability

Not applicable.
